# Association Between the Consumption of Sugar-Sweetened Beverages and High-Caffeine Drinks and Self-Reported Mental Health Conditions Among Korean Adolescents

**DOI:** 10.3390/nu17162652

**Published:** 2025-08-15

**Authors:** Seung Jae Lee, Yeseul Na, Kyung Won Lee

**Affiliations:** 1 Department of Food Science and Nutrition, Yongin University, Yongin 17092, Republic of Korea; sjlee@yongin.ac.kr; 2 Department of Food and Nutrition, Honam University, Gwangju 62399, Republic of Korea; 2022053@honam.ac.kr; 3 Department of Home Economics Education, Korea National University of Education, Cheongju 28173, Republic of Korea

**Keywords:** high-caffeine drink, Korea Youth Risk Behavior Survey, Korean adolescents, mental health, sugar-sweetened beverage

## Abstract

**Background/Objectives**: The rising prevalence of mental health disorders among adolescents underscores the need for early intervention. Of concern is the increasing consumption of sugar-sweetened beverages (SSBs) and high-caffeine drinks, which may adversely affect adolescent mental health. This study examined their association with stress perception, depressive symptoms, suicidal ideation, and loneliness among Korean adolescents. **Methods**: Data were derived from the 2022 Korea Youth Risk Behavior Survey, a nationally representative dataset comprising 51,850 Korean adolescents aged 12–18 years. Beverage consumption frequency was categorized into three groups: none, 1–4 times per week, and ≥5–6 times per week. Logistic regression models were employed to estimate adjusted odds ratios (AORs) and 95% confidence intervals (CIs) for mental health conditions, adjusting for potential confounders. **Results**: Compared with non-consumers, adolescents who consumed SSBs ≥5–6 times per week exhibited significantly higher odds of stress perception (AOR 1.35, 95% CI 1.24–1.46), depressive symptoms (AOR 1.32, 95% CI 1.19–1.46), suicidal ideation (AOR 1.23, 95% CI 1.09–1.39), and loneliness (AOR 1.44, 95% CI 1.28–1.61). Similarly, frequent consumption of high-caffeine drinks (≥5–6 times per week) was associated with increased odds of stress perception (AOR 2.13, 95% CI 1.99–2.29), depressive symptoms (AOR 1.75, 95% CI 1.62–1.88), suicidal ideation (AOR 2.04, 95% CI 1.86–2.24), and loneliness (AOR 1.72, 95% CI 1.59–1.87). **Conclusions**: Frequent consumption of SSBs and high-caffeine drinks is significantly associated with adverse mental health outcomes among Korean adolescents. Given the increasing intake of these beverages, targeted public health interventions are warranted to mitigate their potential negative impact on adolescent mental well-being.

## 1. Introduction

Adolescence presents a critical period characterized by rapid physical, emotional, and social development, during which lifestyle habits established can have a lasting impact on long-term health outcomes [1,2]. Mental health significantly affects adolescents’ academic performance, interpersonal relationships, and quality of life [3,4,5]. In Korea, mental health diagnoses among youth have surged, with anxiety up 93.1% and depression 75.8% between 2018 and 2023 [6], underscoring the urgent need for early intervention.

Recent Korean studies have identified dietary and behavioral factors influencing adolescent mental health. Fast food consumption has been associated with an increased risk of depression, while fruits and vegetables have exhibited a protective effect [7]. Irregular breakfast and high sugar-sweetened beverage (SSB) or fast food consumption have been associated with anxiety disorder [8], and frequent viewing of Mukbang (online eating shows) and Cookbang (online cooking shows) is associated with stress and depressive symptoms [9]. Solitary dining, particularly at dinner, also raises mental health issues among adolescents [10].

A concerning trend among Korean adolescents is the rising consumption of SSBs and high-caffeine drinks, particularly energy drinks [11]. These beverages may disrupt glucose regulation, sleep, and the nervous system, thereby potentially exacerbating mental health conditions. SSBs have been implicated in insulin resistance and alterations in neurotransmitter function [12,13], while high-caffeine drinks have been associated with heightened anxiety, insomnia, and cumulative fatigue [14,15].

While direct research on the relationship between SSB and energy drink consumption and mental health outcomes among Korean adolescents is limited, a growing body of international evidence underscores the significance of this association. Studies conducted in the United States, Canada, and various European countries have consistently reported that high consumption of SSBs and energy drinks is linked to elevated risks of depression, anxiety, and academic performance among adolescents [16,17,18,19,20]. Moreover, recent meta-analyses suggest that excessive intake of caffeinated or sugar-rich beverages may compromise mental well-being by disrupting sleep patterns and increasing systemic inflammation [21,22,23,24]. Despite these findings, investigations into the psychological effects of these beverages on Korean adolescents remain scarce. Most existing studies have focused on physical health outcomes [25,26,27,28] or Western populations [24,29,30], limiting their applicability to Korean adolescents. Given the increasing consumption of SSBs and high-caffeine beverages in this demographic, it is important to assess whether similar mental health risks are evident. Furthermore, identifying relevant sociodemographic and behavioral correlates may inform culturally appropriate public health interventions. This study is conceptually grounded in the biopsychosocial model of health, which explains mental health outcomes as the result of interactions between biological, psychological, and social factors. This framework supports a multifactorial exploration of how beverage consumption habits may be linked to adolescents’ mental health.

In this study, we aimed to evaluate the mental health status of Korean adolescents and investigate the association between SSB and high-caffeine drink consumption frequency and mental health outcomes. Specifically, the research addresses two main questions: (1) What factors influence the consumption of SSBs and high-caffeine drinks among Korean adolescents? and (2) How are the consumption frequencies of these beverages associated with various adverse mental health outcomes in this population? The findings will support evidence-based strategies and inform public health policies to enhance adolescent mental well-being.

## 2. Materials and Methods

### 2.1. Data Source and Study Population

This study used data from the 2022 Korean Youth Risk Behavior Survey (KYRBS), a nationally representative annual survey conducted by the Korea Disease Control and Prevention Agency (KDCA) since 2005 to monitor adolescent health behaviors [31]. Targeting students from the first year of middle school through the third year of high school, the KYRBS employs a two-stage stratified cluster sampling method to ensure national representativeness. It collects comprehensive data on dietary habits, smoking, alcohol consumption, physical activity, and mental health. The KYRBS is a school-based, self-administered, anonymous online survey conducted during regular class hours. All responses were self-reported. The methodology and implementation details of the KYRBS have been extensively documented in previous studies [32].

For the present study, data from 51,850 Korean adolescents (26,397 boys and 25,453 girls) were analyzed. All study procedures followed institutional guidelines with written informed consent, and ethical approval was obtained from the Institutional Review Board of the Korea National University of Education (IRB No. KNUE-202408-BM-0488-01).

### 2.2. Consumption of SSBs and High-Caffeine Drinks

SSB and high-caffeine drink consumption was assessed using self-reported questionnaire items from the KYRBS Dietary Habits section. SSB consumption frequency was evaluated with the question, “During the past 7 days, how often did you consume SSBs (such as carbonated drinks, sports drinks, fruit juices, or sweetened milk)?” Similarly, high-caffeine drink consumption was assessed using the question, “During the past 7 days, how often did you consume high-caffeine drinks (such as energy drinks, coffee, or coffee-based beverages)?” Based on the responses, participants were categorized into three groups: “none,” “1–4 times per week,” and “≥5–6 times per week.”

### 2.3. General Characteristics and Health-Related Behaviors

Collected demographic variables included sex (boys or girls), age (years), school level (middle school or high school), residential area (metropolitan, small- to medium-sized city, or rural), household economic status (low, middle, or high), residential status (living with family or not living with family), and self-reported academic performance (low, middle, or high). Health-related behavior variables included regular physical activity (yes or no), smoking status (yes or no), alcohol consumption (yes or no), weight status (underweight, normal weight, overweight, or obese; classified according to the 2017 Korean National Growth Charts [33]), and sleep duration (<5, 5–6, 6–7, 7–8, or ≥8 h). Additionally, dietary behaviors assessed included experience with nutrition education (yes or no), breakfast skipping (yes or no), and adequate water intake (yes or no). Adequate water intake was defined as consuming ≥5 cups (1 L) per day, whereas inadequate intake was classified as consuming <5 cups per day.

### 2.4. Mental Health Conditions

Adolescents’ mental health status was assessed using KYRBS items on stress, depression, suicidal ideation, and loneliness. Stress perception was determined based on responses to the question, “How much stress do you usually feel?” Adolescents who reported “very much” or “much” stress were classified as experiencing stress. Depressive symptoms were defined as having felt “so sad or hopeless for at least two consecutive weeks in the past 12 months that daily activities were disrupted.” Participants who responded ‘yes’ were categorized as experiencing depressive symptoms. Suicidal ideation was assessed using the question, “Have you seriously considered suicide in the past 12 months?” Adolescents who answered “yes” were classified as having suicidal ideation. Loneliness was evaluated based on the question, “How often have you felt lonely in the past 12 months?” Participants who responded “often” or “always” were considered to have experienced loneliness.

### 2.5. Statistical Analyses

All statistical analyses were conducted using SAS version 9.4 (SAS Institute, Inc., Cary, NC, USA). Following the analytical guidelines of the KYRBS, Proc Survey procedures were employed to account for the complex sampling design by incorporating sample weights, strata, and primary sampling units [31]. The results were reported as frequencies and weighted percentages for categorical variables and as means with standard errors for continuous variables. Differences in general characteristics, health-related behaviors, nutrient intake, and dietary behaviors according to the frequency of SSB and high-caffeine drink consumption were analyzed using chi-square tests. To assess associations between SSB and high-caffeine drink consumption frequency and mental health outcomes—including stress perception, depressive symptoms, suicidal ideation, and loneliness—multiple logistic regression models were applied, adjusting for covariates, including sex, school level, residential area, household economic status, residential status, academic performance, regular physical activity, smoking, alcohol consumption, weight status, sleep duration, breakfast skipping, adequate water intake, and experience of nutrition education. Additionally, to explore potential differences by educational stage, stratified analyses were conducted separately for middle school and high school students. This stratification allowed for the examination of whether the associations between beverage consumption frequency and mental health outcomes varied by school level. Adjusted odds ratios (AORs) and 95% confidence intervals (CIs) were calculated. Statistical significance was defined as a two-tailed *p*-value of <0.05 in all analyses.

## 3. Results

Table 1 presents the general characteristics of the study population. Among the 51,850 Korean adolescents in the 2022 KYRBS, 6.6% (*n* = 3427) reported no SSB consumption, 63.8% (*n* = 33,245) consumed SSBs 1–4 times per week, and 29.6% (*n* = 15,178) consumed SSBs ≥5–6 times per week. For high-caffeine drink consumption, 51.30% (*n* = 26,876) reported no consumption, 38.5% (*n* = 19,892) consumed them ≥1–4 times per week, and 10.2% (*n* = 5082) consumed them ≥5–6 times per week. The sample was nearly evenly distributed by sex, with 51.6% boys and 48.4% girls, and had a mean age of 15.2 years. In terms of education level, 51.6% of the participants were middle school students, while 48.4% were in high school. Regarding residence, 52.9% lived in small to medium cities, 41.5% in metropolitan areas, and 5.6% in rural regions. The reported household economic status was predominantly middle (46.0%) and high (43.3%) income. Subjective academic performance was categorized as high in 38.8% of the participants, middle in 30.0%, and low in 31.2%.

**Figure 1:**
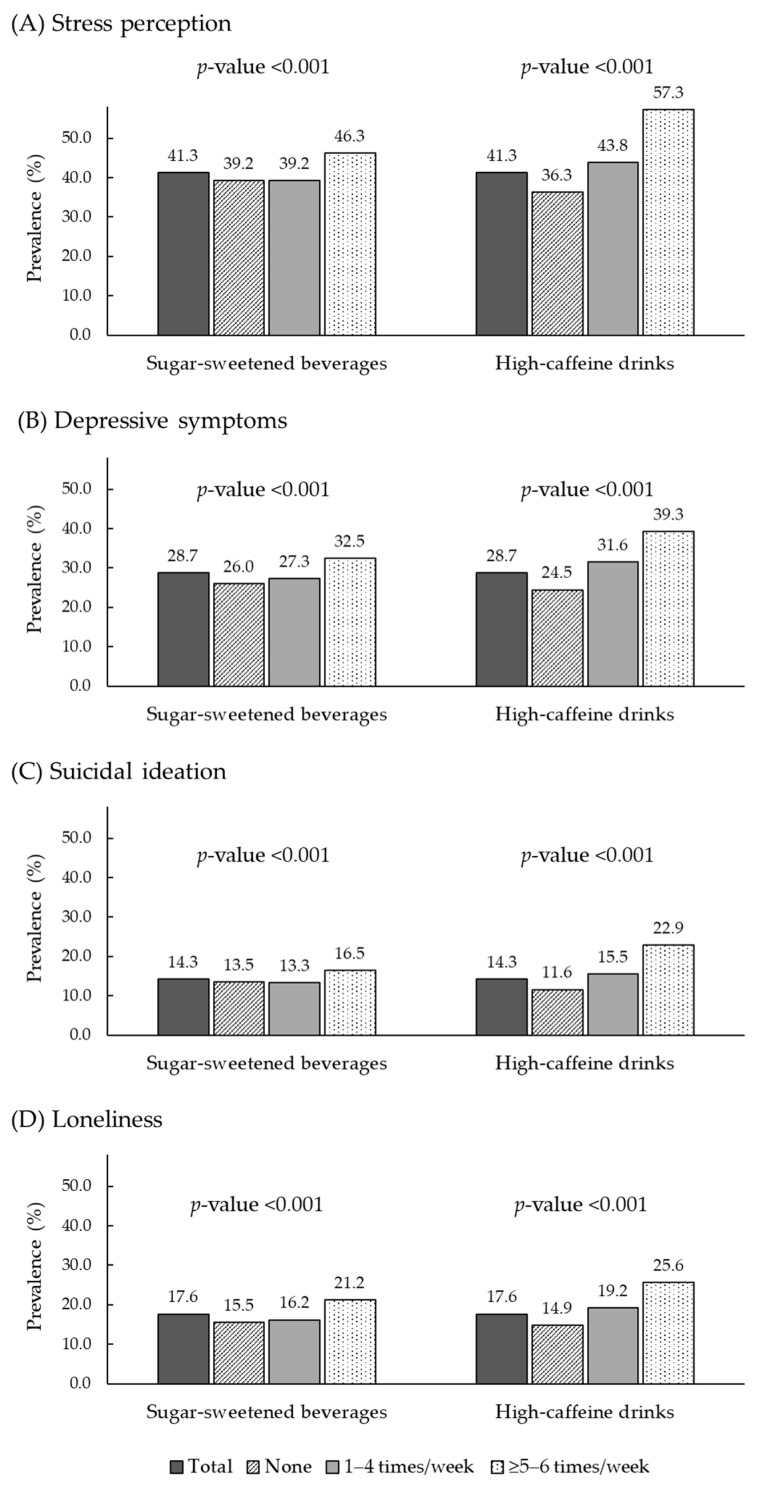
Proportion of poor mental health outcomes according to the consumption frequency of sugar-sweetened beverages and high-caffeine drinks. *p*-values were obtained from the chi-square test to examine differences in the distribution of variables according to the consumption frequency of SSBs and high-caffeine drinks.

Figure 1 illustrates the prevalence of adverse mental health outcomes according to SSB and high-caffeine drink consumption frequency. Significant differences in stress perception, depressive symptoms, suicidal ideation, and feelings of loneliness were found across consumption levels (all *p*-value < 0.001). Overall, 41.3% reported stress perception and 28.7% reported depressive symptoms. These were most prevalent among those consuming SSBs (46.3%) or high-caffeine drinks (57.3%) ≥5–6 times per week, with depressive symptoms highest at 32.5% and 39.3%, respectively. Suicidal ideation (14.3%) and loneliness (17.6%) also peaked in the ≥5–6 times per week group (SSBs: 16.5% and 21.2%; high-caffeine drinks: 22.9% and 25.6%).

The general characteristics of the study population according to SSB and high-caffeine drink consumption frequency are presented in Table S1. SSB consumption was significantly associated with sex, school level, household economic status, and academic performance (all *p*-values < 0.01). High-caffeine drink consumption was significantly associated with school level, residential area, household economic status, and academic performance (all *p*-values < 0.001).

Table S2 summarizes health behaviors and dietary characteristics by SSB and high-caffeine drink consumption frequency. Both SSB and high-caffeine drink consumption were significantly associated with sleep duration, current smoking and alcohol consumption, weight status, regular physical activity, breakfast skipping, adequate water intake, and prior exposure to nutrition education (all *p*-values < 0.001).

Table 2 presents the factors associated with frequent beverage consumption. Adolescents who were boys, in high school, lived in cities, had lower academic performance, slept less, smoked, consumed alcohol, were overweight or obese, were physically inactive, skipped breakfast, or had inadequate water intake were more likely to consume SSBs ≥5–6 times per week (all *p*-values < 0.05). Similarly, high-caffeine drink consumption (≥5–6 times/week) was more prevalent among high school students, those living in cities, and those with shorter sleep duration, current smoking or drinking habits, overweight or obesity, low physical activity, breakfast skipping, and inadequate water intake (all *p*-values < 0.05).

Table 3 presents the association between SSB consumption and mental health outcomes. Adolescents consuming SSBs ≥5–6 times per week exhibited significantly higher odds of stress perception, depressive symptoms, suicidal ideation, and loneliness than non-consumers. When stratified by school level, frequent SSB consumption (≥5–6 times per week) was significantly associated with stress perception, depressive symptoms, and feelings of loneliness among both middle and high school students. Additionally, high school students who consumed SSBs frequently showed elevated odds of suicidal ideation than non-consumers.

Table 4 presents the AORs ratios for poor mental health outcomes by high-caffeine drink consumption. Compared to non-consumers, adolescents consuming high-caffeine drinks 1–4 times or ≥5–6 times per week showed significantly higher odds of all assessed poor mental health outcomes, including stress perception, depressive symptoms, suicidal ideation, and loneliness. This pattern was consistent across both middle and high school groups, demonstrating a dose–response relationship between frequency of consumption and adverse mental health outcomes.

## 4. Discussion

This cross-sectional study analyzed 2022 KYRBS to assess mental health disorder prevalence among Korean adolescents aged 12–18 years. This study investigated associations between the frequency of SSB and high-caffeine drink consumption and the prevalence of stress perception, depressive symptoms, suicidal ideation, and loneliness. Adolescents consuming SSBs ≥5–6 times per week were more likely to report stress perception, depressive symptoms, suicidal ideation, and loneliness. Furthermore, those consuming high-caffeine drinks at a similar frequency also showed higher odds of stress perception, depressive symptoms, suicidal ideation, and loneliness.

The study identified a substantial prevalence of self-reported mental health concerns among Korean adolescents, including stress perception (41.3%), depressive symptoms (28.7%), suicidal ideation (14.3%), and loneliness (17.6%). A stratified analysis by school level revealed a trend toward a higher prevalence of all assessed mental health concerns—except suicidal ideation—among high school students compared to their middle school counterparts. This pattern aligns with the existing literature highlighting the increasing psychological burden experienced by Korean high school students, which is attributed to heightened academic competition, elevated expectations for academic performance, and a reduced capacity to manage stress [34,35].

SSBs, high in sugar or high-fructose corn syrup, are major sources with minimal nutritional benefits. Frequent SSB consumption has been linked in previous studies to increased risks of obesity, type 2 diabetes, and all-cause mortality in both adolescents and adults [36,37,38]. While physical health risks are well-documented, the mental health impact—particularly in adolescents—remains underexplored. Prior research, predominantly focused on adults in Korea, the United States, and China, suggests an association between SSB consumption and higher depression incidence [39,40,41]. A meta-analysis by Hu et al. [24] corroborated this relationship, demonstrating increased depression risk with SSB consumption equivalent to two cups of cola daily. However, only one study in that analysis included children and adolescents. Recently, more research has begun to investigate this association in adolescents. A large-scale global study of 105,061 adolescents identified a significant association between carbonated soft drink consumption and suicidal attempts, with variations by income level [42]. Similarly, studies in China also reported a positive association between SSB intake and depressive symptoms in children and adolescents [18,43].

Although the observed associations between frequent SSB consumption and adverse mental health outcomes were statistically significant, the corresponding AORs (ranging from approximately 1.2 to 1.6) indicate small to moderate effect sizes. These findings, while important from a population health perspective, should be interpreted cautiously when considering their practical significance at the individual level. Nevertheless, even modest increases in risk may translate into a meaningful public health impact given the high prevalence of both SSB consumption and adolescent mental health challenges in Korea.

While the precise mechanisms underlying the association between SSB consumption and adverse mental health outcomes require further elucidation, several biological pathways are plausible. One potential mechanism involves the glycemic effects of SSBs. The high sugar content of these beverages rapidly raises blood glucose levels, triggering insulin spikes. Over time, chronic fluctuations can lead to insulin resistance, which is increasingly linked to depression and suicidal behaviors [44,45]. Another potential pathway involves hypothalamic–pituitary–adrenal axis dysregulation—blood sugar swings may activate this stress–response system [46], elevating cortisol and impairing hippocampal function, both associated with depression, anxiety, and other mental health disorders [47,48,49]. Excess sugar intake may also lower brain-derived neurotrophic factor (BDNF), which is essential for neuronal health. Reduced BDNF has been tied to hippocampal atrophy, cognitive decline, and a greater risk of mood disorders [50]. This hypothesis is supported by experimental animal studies demonstrating significantly reduced BDNF expression in rodents fed high-fat, high-sugar diets compared to those consuming high-fat diets alone [51,52].

The present study identified a significant, dose-dependent association between high-caffeine drink consumption and adverse mental health outcomes in Korean adolescents, with stronger effects in middle school students. Importantly, a linear trend was observed across consumption frequency groups, with adolescents consuming high-caffeine drinks ≥5–6 times per week consistently exhibiting higher odds of all adverse mental health outcomes compared to both 1–4 times/week consumers and non-consumers. This pattern suggests a potential dose–response relationship, further substantiating the plausibility of a link between consumption frequency and psychological burden.

These findings align with prior research demonstrating a significant relationship between the consumption of high-caffeine drinks, including energy drinks, and the incidence of depression, anxiety, emotional disorders, and suicidal behaviors in adolescents [53,54]. High-caffeine drinks in this study included energy drinks, coffee, and coffee-based beverages, with caffeine content varying by product type in South Korea. On average, one serving contained 80.2 mg of caffeine in energy drinks, 132.0 mg in specialty coffee shop coffee, and 47.0 mg in coffee-flavored milk. In response to the increasing consumption of high-caffeine drinks among Korean adolescents, the Ministry of Food and Drug Safety recommends a caffeine limit of 2.5 mg per kg of body weight for children and adolescents [55]. Nonetheless, Korean adolescents consume an average of 16.2 mg, with over 50% coming from carbonated beverages, including energy drinks [55]. The mental health impact may result from caffeine’s interaction with sugars and stimulants in these beverages, affecting the brain [56,57]. Though often used to boost focus or mood [58,59], excessive intake can overstimulate the nervous system, disrupt sleep, and lead to insomnia [60,61], which contributes to anxiety, agitation, and nervousness [23,62,63,64]. Additionally, caffeine’s addictive nature may promote habitual use and worsen mental health symptoms over time [65]. However, the observed associations between high-caffeine drink consumption and mental health indicators in this study do not imply a direct causal relationship. Adolescents experiencing psychological distress may be more likely to consume these beverages as a means of mood regulation or to increase alertness. Additionally, unmeasured confounding factors related to lifestyle or environment may influence both beverage consumption and mental health status.

Notably, the findings of this study suggest that middle school students may be more susceptible to the mental health effects of frequent high-caffeine drink consumption. When consumed ≥5–6 times per week, the odds of perceived stress, depressive symptoms, suicidal ideation, and loneliness were significantly higher in middle school students compared to high school students. Several potential factors may explain this. Caffeine sensitivity varies by genetic predisposition, age, body weight, and overall health status [66], with age playing a critical role due to metabolic and liver function differences [67]. A national study of children and adolescents aged 6–19 years found a stronger association between caffeine exposure and obesity in younger children aged 6–11 years than in adolescents aged 12–19 years, implying greater vulnerability to caffeine’s physiological and possibly psychological effects [68]. Furthermore, mental health challenges in Korean adolescents tend to increase with age due to growing academic pressure, reduced coping capacity, and shifting social support [69,70,71]. As these stressors intensify, the relative impact of caffeine intake on mental health may appear to diminish with age.

This study has several limitations that should be acknowledged. First, the cross-sectional design of this study prevents causal relationships between the consumption of SSBs and high-caffeine drinks and poor mental health outcomes. Second, reliance on self-reported data on SSB and high-caffeine drink consumption, as well as the presence of adverse mental health conditions, may introduce recall bias and systematic reporting errors. Third, the assessment of key variables, including mental health status and health-related behaviors such as alcohol use, smoking, and physical activity, was based on overly simplistic measures rather than validated psychological or behavioral scales. This methodological limitation may have reduced the precision and depth of interpretation. Fourth, while consumption frequency was assessed, variations in sugar and caffeine content within beverage types were not considered. Future research should explore how these differences affect mental health outcomes. Despite these limitations, this is the latest study to investigate the association between the frequency of SSB and high-caffeine drink consumption and multiple mental health outcomes in a nationally representative sample of Korean adolescents.

## 5. Conclusions

This study demonstrates a significant association between frequent consumption of SSBs and high-caffeine drinks and adverse mental health outcomes among Korean adolescents. Consuming SSBs or high-caffeine drinks ≥5–6 times per week was linked to a higher likelihood of stress perception, depressive symptoms, suicidal ideation, and loneliness. Given the rising intake of these beverages and the increasing mental health concerns in this population, these findings highlight the need for evidence-based public health interventions. Dietary educational programs promoting reduced consumption of SSB and high-caffeine beverages and encouraging healthier beverage consumption habits from an early age are essential. Future research should employ prospective designs to explore causality and evaluate targeted intervention strategies aimed at improving adolescent dietary behaviors and psychological well-being. These findings provide a foundation for evidence-based guidelines and policies that support the mental well-being of Korean adolescents and contribute to long-term public health advancements.

## Figures and Tables

**Table 1:** General characteristics of the study participants.

Variable	Total n (Wt’d %)
Total	51,850 (100.00)
Consumption frequency of SSBs	
None	3427 (6.56)
1–4 times/week	33,245 (63.84)
≥5–6 times/week	15,178 (29.60)
Consumption frequency of high-caffeine drinks	
None	26,876 (51.30)
1–4 times/week	19,892 (38.53)
≥5–6 times/week	5082 (10.17)
Sex	
Boys	26,397 (51.56)
Girls	25,453 (48.44)
Age	15.20 ± 0.03
School level	
Middle school	28,015 (51.64)
High school	23,835 (48.36)
Residential area	
Metropolitan	22,212 (41.54)
Small- to medium-sized city	25,814 (52.88)
Rural	3824 (5.58)
Household economic status	
Low	5816 (10.71)
Middle	24,146 (46.01)
High	21,888 (43.28)
Subjective academic performance	
Low	16,313 (31.18)
Middle	15,484 (30.02)
High	20,053 (38.80)

Wt’d %, weighted %. Data are presented as frequencies (weighted %) or means ± standard errors (SEs).

**Table 2:** Factors associated with consumption of sugar-sweetened beverages and high-caffeine drinks (≥5–6 times per week) in Korean adolescents.

Variable	Sugar-Sweetened Beverages	High-Caffeine Drinks
	AOR (95% CI)	AOR (95% CI)
Sex		
Boys	1.44 (1.38–1.51) ^(1)^	0.97 (0.90–1.05)
Girls	Ref.	Ref.
School level		
Middle school	Ref.	Ref.
High school	1.13 (1.07–1.19)	1.56 (1.44–1.69)
Residential area		
Metropolitan	1.18 (1.07–1.31)	1.37 (1.17–1.60)
Small- to medium-sized city	1.18 (1.06–1.30)	1.32 (1.14–1.54)
Rural	Ref.	Ref.
Household economic status		
Low	1.00 (0.93–1.07)	1.03 (0.92–1.14)
Middle	0.96 (0.92–1.00)	0.83 (0.77–0.90)
High	Ref.	Ref.
Residential status		
Living with family	Ref.	Ref.
Not living with family	1.05 (0.95–1.17)	1.03 (0.87–1.22)
Subjective academic performance		
Low	1.10 (1.05–1.17)	0.94 (0.86–1.02)
Middle	0.97 (0.92–1.03)	0.93 (0.86–1.01)
High	Ref.	Ref.
Sleep duration		
Less than 5 h	1.48 (1.38–1.59)	3.47 (3.10–3.89)
5–6 h	1.30 (1.22–1.39)	2.56 (2.29–2.86)
6–7 h	1.11 (1.05–1.19)	1.56 (1.41–1.73)
7–8 h	1.07 (1.00–1.13)	1.16 (1.04–1.30)
More than 8 h	Ref.	Ref.
Current smoking		
Yes	1.43 (1.29–1.59)	1.70 (1.49–1.94)
No	Ref.	Ref.
Current drinking		
Yes	1.30 (1.21–1.39)	1.53 (1.39–1.68)
No	Ref.	Ref.
Weight status		
Underweight	0.94 (0.86–1.04)	1.08 (0.96–1.22)
Normal weight	Ref.	Ref.
Overweight	1.21 (1.13–1.30)	1.19 (1.07–1.32)
Obesity	1.36 (1.23–1.49)	1.20 (1.08–1.34)
Regular physical activity		
Yes	Ref.	Ref.
No	1.14 (1.09–1.19)	1.10 (1.03–1.18)
Breakfast skipping		
Yes	1.14 (1.09–1.19)	1.22 (1.14–1.30)
No	Ref.	Ref.
Adequate water intake		
Yes	Ref.	Ref.
No	1.30 (1.24–1.36)	1.12 (1.04–1.20)
Nutritional education		
Yes	0.99 (0.95–1.04)	0.94 (0.88–1.01)
No	Ref.	Ref.

AOR, adjusted odds ratio; 95% CI, 95% confidence interval. ^(1)^ Multiple logistic regression analysis was performed to estimate the odds for the consumption of sugar-sweetened beverages or high-caffeine drinks ≥5–6 times/week for the study participants: the analytic model was adjusted for sex, school level, residential area, household economic status, residential status, academic performance, regular physical activity, smoking, drinking, weight status, sleep duration, skipping breakfast, adequate water intake, and experience of nutrition education.

**Table 3:** Association of sugar-sweetened beverage consumption frequency with poor mental health conditions among Korean adolescents.

	Total (*n* = 51,850)				Middle School (*n* = 28,015)				High School (*n* = 23,835)			
	None	1–4 Times/Week	≥5–6 Times/Week	*p*-Value	None	1–4 Times/Week	≥5–6 Times/Week	*p*-Value	None	1–4 Times/Week	≥5–6 Times/Week	***p*-Value**
	(*n* = 3427)	(*n* = 33,245)	(*n* = 15,178)		(*n* = 1954)	(*n* = 18,464)	(*n* = 7597)		(*n* = 1473)	(*n* = 14,781)	(*n* = 7581)	
		AOR (95% CI)	AOR (95% CI)			AOR (95% CI)	AOR (95% CI)			AOR (95% CI)	AOR (95% CI)	
Stress Perception	1.00	1.00 (0.92–1.09) ^(1)^	1.35 (1.24–1.46)	<0.0001	1.00	0.96 (0.86–1.07)	1.25 (1.12–1.41)	<0.0001	1.00	1.05 (0.93–1.18)	1.43 (1.27–1.60)	<0.0001
Depressive symptoms	1.00	1.05 (0.96–1.15)	1.32 (1.19–1.46)	<0.0001	1.00	0.99 (0.87–1.12)	1.26 (1.09–1.46)	<0.0001	1.00	1.13 (0.99–1.29)	1.38 (1.20–1.59)	<0.0001
Suicidal ideation	1.00	0.97 (0.86–1.09)	1.23 (1.09–1.39)	<0.0001	1.00	0.93 (0.80–1.08)	1.15 (0.98–1.35)	<0.0001	1.00	1.02 (0.84–1.24)	1.33 (1.09–1.62)	<0.0001
Loneliness	1.00	1.04 (0.94–1.16)	1.44 (1.28–1.61)	<0.0001	1.00	0.95 (0.83–1.10)	1.31 (1.13–1.53)	<0.0001	1.00	1.16 (0.99–1.37)	1.61 (1.36–1.91)	<0.0001

AOR, adjusted odds ratio; 95% CI, 95% confidence interval. ^(1)^ Multiple logistic regression analysis was performed to estimate the odds for poor mental health conditions for the study participants: the analytic model was adjusted for sex, school level, residential area, household economic status, residential status, academic performance, regular physical activity, smoking, drinking, weight status, sleep duration, skipping breakfast, adequate water intake, and experience of nutrition education.

**Table 4:** Association of high-caffeine drink consumption frequency with poor mental health conditions among Korean adolescents.

	Total (*n* = 51,850)				Middle School (*n* = 28,015)				High School (*n* = 23,835)			
	None	1–4 Times/Week	≥5–6 Times/Week	*p*-Value	None	1–4 Times/Week	≥5–6 Times/Week	*p*-Value	None	1–4 Times/Week	≥5–6 Times/Week	*p*-Value
	(*n* = 26,876)	(*n* = 19,892)	(*n* = 5082)		(*n* = 16,192)	(*n* = 9869)	(*n* = 1954)		(*n* = 10,684)	(*n* = 10,023)	(*n* = 3128)	
		AOR (95% CI)	AOR (95% CI)			AOR (95% CI)	AOR (95% CI)			AOR (95% CI)	AOR (95% CI)	
Stress Perception	1.00	1.30 (1.25–1.36) ^(1)^	2.13 (1.99–2.29)	<0.0001	1.00	1.35 (1.27–1.43)	2.35 (2.12–2.60)	<0.0001	1.00	1.23 (1.16–1.31)	1.96 (1.78–2.15)	<0.0001
Depressive symptoms	1.00	1.32 (1.26–1.38)	1.75 (1.62–1.88)	<0.0001	1.00	1.41 (1.32–1.52)	1.91 (1.70–2.16)	<0.0001	1.00	1.20 (1.12–1.28)	1.58 (1.44–1.73)	<0.0001
Suicidal ideation	1.00	1.32 (1.24–1.41)	2.04 (1.86–2.24)	<0.0001	1.00	1.38 (1.26–1.51)	2.42 (2.12–2.76)	<0.0001	1.00	1.24 (1.13–1.35)	1.74 (1.54–1.98)	<0.0001
Loneliness	1.00	1.27 (1.20–1.35)	1.72 (1.59–1.87)	<0.0001	1.00	1.36 (1.25–1.48)	2.06 (1.84–2.32)	<0.0001	1.00	1.18 (1.08–1.27)	1.50 (1.34–1.68)	<0.0001

AOR, adjusted odds ratio; 95% CI, 95% confidence interval. ^(1)^ Multiple logistic regression analysis was performed to estimate the odds for poor mental health conditions for the study participants: the analytic model was adjusted for sex, school level, residential area, household economic status, residential status, academic performance, regular physical activity, smoking, drinking, weight status, sleep duration, skipping breakfast, adequate water intake, and experience of nutrition education.

## Data Availability

The data (2022 KYRBS) presented in this study are available at https://www.kdca.go.kr/yhs/yhs/main.do (accessed on 8 January 2025).

## Supplementary Materials

The following supporting information can be downloaded at https://www.mdpi.com/article/10.3390/nu17162652/s1: Table S1: General characteristics of study participants according to sugar-sweetened beverages and high-caffeine drink consumption frequency among Korean adolescents; Table S2: Health-related and dietary behaviors according to sugar-sweetened beverages and high-caffeine drink consumption frequency among Korean adolescents.

## Abbreviations

The following abbreviations are used in this manuscript:

SSB	Sugar-sweetened beverage
KYRBS	Korean Youth Risk Behavior Survey
KDCA	Korea Disease Control and Prevention Agency
AOR	Adjusted odds ratio
CI	Confidence interval
BDNF	Brain-derived neurotrophic factor
