# Supplementary material for: Association Between the Consumption of Sugar-Sweetened Beverages and High-Caffeine Drinks and Self-Reported Mental Health Conditions Among Korean Adolescents
Source: Nutrients. 2025 Aug 15;17(16):2652. doi: 10.3390/nu17162652 (PMC12389327; doi:10.3390/nu17162652)
Supplement: Supplementary file 1 [file nutrients-17-02652-s001.zip › nutrients-3784848-supplementary.pdf]

Table S1. General characteristics of study participants according to sugar-sweetened beverages and high-caffeine drink consumption frequency among Korean adolescents.

Variables	Total	Sugar-sweetened beverages				High-caffeine drinks			
		None	1–4 times/week	≥5–6 times/week	*p*-value[1)]	None	1–4 times/week	≥5–6 times/week	*p*-value
	n (Wt'd %)	*n* (Wt'd %)	*n* (Wt'd %)	*n* (Wt'd %)		*n* (Wt'd %)	*n* (Wt'd %)	*n* (Wt'd %)	
**Sex**					<0.0001				0.5919
Men	26,397 (51.56)	1,613 (6.17)	16,189 (60.99)	8,595 (32.84)		13,595 (51.00)	10,192 (38.73)	2,610 (10.27)	
Women	25,453 (48.44)	1,814 (6.98)	17,056 (66.88)	6,583 (26.14)		13,281 (51.61)	9,700 (38.31)	2,472 (10.07)	
**School level**					<0.0001				<0.0001
Middle school	28,015 (51.64)	1,954 (6.94)	18,464 (65.87)	7,597 (27.19)		16,192 (57.93)	9,869 (35.10)	1,954 (6.96)	
High school	23,835 (48.36)	1,473 (6.16)	14,781 (61.68)	7,581 (32.16)		10,684 (44.21)	10,023 (42.19)	3,128 (13.60)	
**Residential area**					0.0516				0.0061
Metropolitan	22,212 (41.54)	1,414 (6.34)	14,249 (63.88)	6,549 (29.78)		11,351 (50.65)	8,563 (38.70)	2,298 (10.65)	
Small-to-medium-sized city	25,814 (52.88)	1,746 (6.68)	16,475 (63.58)	7,593 (29.74)		13,517 (51.71)	9,815 (38.27)	2,482 (10.02)	
Rural	3,824 (5.58)	267 (7.05)	2,521 (66.09)	1,036 (26.86)		2,008 (52.18)	1,514 (39.73)	302 (8.09)	
**Household economic status**					0.0022				<0.0001
Low	5,816 (10.71)	401 (7.09)	3,618 (61.44)	1,797 (31.47)		2,836 (48.79)	2,285 (38.84)	695 (12.37)	
Middle	24,146 (46.01)	1,555 (6.37)	15,614 (64.55)	6,977 (29.08)		12,730 (52.18)	9,265 (38.59)	2,151 (9.23)	
High	21,888 (43.28)	1,471 (6.63)	14,013 (63.69)	6,404 (29.68)		11,310 (50.98)	8,342 (38.39)	2,236 (10.63)	
**Subjective academic performance**					<0.0001				<0.0001
Low	16,313 (31.18)	1,012 (6.25)	10,078 (61.73)	5,223 (32.02)		7,944 (48.48)	6,625 (40.60)	1,744 (10.92)	
Middle	15,484 (30.02)	1,033 (6.55)	10,145 (65.21)	4,306 (28.24)		7,975 (50.68)	6,058 (39.54)	1,451 (9.78)	
High	20,053 (38.80)	1,382 (6.82)	13,022 (64.48)	5,649 (28.70)		10,957 (54.04)	7,209 (36.08)	1,887 (9.88)	

Wt'd %, weighted %. Data are presented as frequencies (weighted %). [1)] *p*-values were calculated using the chi-squared test.

Table S2. Health-related and dietary behaviors according to sugar-sweetened beverages and high-caffeine drink consumption frequency among Korean adolescents.

Variables	Total	Sugar-sweetened beverages				High-caffeine drinks			
		None	1–4 times/week	≥5–6 times/week	*p*-value[1)]	None	1–4 times/week	≥5–6 times/week	*p*-value
	n (Wt'd %)	*n* (Wt'd %)	*n* (Wt'd %)	*n* (Wt'd %)		*n* (Wt'd %)	*n* (Wt'd %)	*n* (Wt'd %)	
**Sleep duration**					<0.0001				<0.0001
Less than 5 hours	6,059 (12.12)	415 (6.83)	3,492 (57.13)	2,152 (36.04)		2,313 (37.29)	2,544 (42.16)	1,202 (20.55)	
5-6 hours	7,403 (14.86)	470 (6.36)	4,499 (61.05)	2,434 (32.59)		3,010 (39.88)	3,260 (44.43)	1,133 (15.70)	
6-7 hours	11,749 (23.25)	675 (5.62)	7,643 (65.03)	3,431 (29.35)		5,751 (48.94)	4,864 (41.26)	1,134 (9.80)	
7-8 hours	12,087 (23.18)	792 (6.45)	7,926 (65.35)	3,369 (28.20)		6,771 (55.98)	4,496 (37.07)	820 (6.95)	
More than 8 hours	14,552 (26.59)	1,075 (7.46)	9,685 (66.12)	3,792 (26.42)		9,031 (62.05)	4,728 (32.46)	793 (5.49)	
**Current smoking**					<0.0001				<0.0001
Yes	2,449 (4.89)	97 (3.89)	1,282 (51.86)	1,070 (44.26)		677 (27.82)	1,209 (49.18)	563 (23.00)	
No	49,401 (95.11)	3,330 (6.70)	31,963 (64.46)	14,108 (28.84)		26,199 (52.50)	18,683 (37.98)	4,519 (9.51)	
**Current drinking**					<0.0001				<0.0001
Yes	6,679 (13.05)	294 (4.47)	3,850 (57.36)	2,535 (38.17)		2,215 (32.70)	3,291 (49.49)	1,173 (17.81)	
No	45,171 (86.95)	3,133 (6.87)	29,395 (64.82)	12,643 (28.31)		24,661 (54.09)	16,601 (36.88)	3,909 (9.03)	
**Weight status**					<0.0001				<0.0001
Underweight	3,947 (7.59)	292 (7.55)	2,371 (59.20)	1,284 (33.25)		2,053 (51.80)	1,448 (36.77)	446 (11.43)	
Normal weight	37,306 (72.43)	2,402 (6.38)	23,818 (63.65)	11,086 (29.97)		19,699 (52.33)	14,192 (38.17)	3,415 (9.50)	
Overweight	4,509 (8.57)	313 (6.68)	3,044 (67.49)	1,152 (25.83)		2,248 (49.06)	1,761 (39.18)	500 (11.76)	
Obesity	6,088 (11.41)	420 (6.96)	4,012 (65.42)	1,656 (27.62)		2,876 (46.10)	2,491 (41.50)	721 (12.40)	
**Regular physical activity**					<0.0001				<0.0001
Yes	19,615 (37.44)	1,294 (6.60)	12,235 (62.14)	6,086 (31.26)		9,884 (49.87)	7,771 (39.84)	1,960 (10.30)	
No	32,235 (62.56)	2,133 (6.54)	21,010 (64.86)	9,092 (28.60)		16,992 (52.15)	12,121 (37.75)	3,122 (10.10)	
**Breakfast skipping**					<0.0001				<0.0001
Yes	20,366 (39.04)	1,220 (5.97)	12,774 (62.34)	6,372 (31.70)		9,765 (47.42)	8,281 (40.79)	2,320 (11.79)	
No	31,484 (60.96)	2,207 (6.94)	20,471 (64.81)	8,806 (28.25)		17,111 (53.78)	11,611 (37.08)	2,762 (9.14)	
**Adequate water intake**					<0.0001				0.0006
Yes	19,208 (36.97)	1,578 (8.12)	12,459 (64.60)	5,171 (27.27)		9,878 (50.92)	7,296 (38.12)	2,034 (10.96)	
No	32,642 (63.03)	1,849 (5.64)	20,786 (63.40)	10,007 (30.96)		16,998 (51.52)	12,596 (38.77)	3,048 (9.71)	
**Nutritional education**					<0.0001				<0.0001
Yes	23,955 (45.35)	1,510 (6.27)	15,636 (64.98)	6,809 (28.75)		12,784 (53.12)	9,058 (37.99)	2,113 (8.89)	
No	27,895 (54.65)	1,917 (6.80)	17,609 (62.90)	8,369 (30.30)		14,092 (49.79)	10,834 (38.98)	2,969 (11.24)	

Wt'd %, weighted %. Data are presented as frequencies (weighted %). [1)] *p*-values were calculated using the chi-squared test.
